# Some Points in the Antiseptic Treatment of Root-Canals

**Published:** 1897-06

**Authors:** J. Morgan Howe


### SOME POINTS IN THE ANTISEPTIC TREATMENT OF ROOT-CANALS.[1]

BY J. MORGAN HOWE, M.D., M.D.S.

OUR friends the bacteriologists have so concentrated our attention upon the germicidal properties of substances that we are in danger of overlooking altogether the antiseptic effects of fillings made of inert or neutral materials. A root-canal that has had a living, or recently devitalized pulp removed from it by means of a clean broach, is the best possible case for immediate filling; and if such a canal is thoroughly filled with any inert and impermeable substance, it is placed in the most perfect and enduring antiseptic condition. That is to say, no germicidal substance, however potent, put into a root to eke out the imperfections of a filling, or mixed with a permeable material, will be so sure of maintaining an antiseptic condition as will a perfectly tight filling, because it keeps everything else out by occupying the space.

The first filling of a root-canal has been attributed to Hudson by Dr. Edward Maynard, who saw the work thirty-five years after it was done, in or about 1807. There were no germicides known as such at that time. The process was a mechanical one, laborious and slow, but all the more likely to be perfect in its results. It was mechanical antisepsis, although the fathers did not know any such word. We have made many advances, but we do not to-day know any other means so effective for preventing the pulpless condition of a root from being a source of danger to the tissues of the socket as the complete filling of the root-canal.

The progress which we cannot withstand is demanding of us today that we should make pulpless root-canals inoffensive, not occasionally, and with much labor, endurance, and expense, but that we shall do it often, quickly, and at a minimum of expense. Witzel, Baume, Loderberg, Herbst, Bödecker, Miller, and others have written on the proposition to impregnate the pulp-tissue with germicides so as to prevent putrefaction, and claims have been made that such treatment has been very successful. We occasionally hear, also, from eminent practitioners that filling roots with cotton saturated with antiseptic liquids is a very effectual means of accomplishing the object. But we know that dead pulps often remain in root-canals for years without treatment, causing no irritation of

---

[1] Read before the New York Institute of Stomatology, March, 1897.

peridental tissues; or, if there is occasional irritation, it frequently passes away without becoming serious, just as often occurs in the case of teeth whose root-canals have been treated. Why may we not conclude, then, that leaving dead pulps in root-canals undisturbed is good treatment? Because we know what bacteriology has taught us, that danger to the periapical tissues is to be attributed to the results of the disorganization of the pulp,—the ptomaines or toxins. We must conclude, therefore, that removing a portion of a dead pulp is better than leaving it all in; that impregnating it with antiseptics must have an influence which lessens or postpones the evil effects that would be likely to follow unretarded putrescence; and that a permeable filling such as cotton, saturated with some antiseptic, is better than no filling, for it occupies a portion of the space that should be entirely taken up, and the potency of the antiseptic agent endures for a variable time. But these are all inferior means, allowable only because complete removal of pulps and thorough filling of canals is difficult, and in some cases impossible, and because temporary treatment is required for special reasons. The principle involved in the filling of root-canals is the removal and exclusion of organic matter. Inert material is all that is required, provided the space is all occupied. The difficulty of doing perfect work and the uncertain results of treatment of periapical tissues has led to the use of permeable material whose interstices are filled with germicides, and to considerations of the ease of removal of root-fillings. The occasional reports that are made of roots that had been filled with cotton, and found inoffensive after a number of years of perfect comfort, are very few relatively to the unreported cases of roots so filled that have caused irritation, inflammation, and abscesses, and occasionally reflex neuralgia for years, and when examined are found in the most offensive condition imaginable. Given an open foramen, and the latter condition would seem certain to result after a time. Absence of recognizable irritations is no criterion of scientific treatment here, for we all recall numbers of teeth which have contained putrescent pulps for years without causing the patient any trouble. We must act on our knowledge of the principles involved in cause and cure. Even temporary treatment of root-canals with cotton saturated with antiseptics and covered with gutta-percha, to remain during good behavior, has its danger in the liability to behave well long enough to be forgotten, and then to have the onset of inflammation occur at the most inopportune time. There is great temptation to busy and to lazy practitioners to do this kind of temporary work, because of the difficulty and, occasionally, the impossibility of doing perfect work in root-canal filling. The patient ought at the least to be informed of the need of more thorough work at a later time. An incident of practice will illustrate from both points of view. I received an early call one morning recently to relieve a patient who had been up all night with toothache. The removal of some gutta-percha from a superior molar where it had not been subjected to attrition enabled me to also remove a cotton root-canal filling that was in a very offensive condition. Relief followed very quickly after such venting. The patient informed me that it must have been so treated as much as nine years ago, and that no serious trouble had occurred before, although occasionally it had been sore for a day or two. Up to the time of severe pain, and the uncovering of the conditions existing, this might have been claimed as successful treatment by both dentist and patient.

It is a consummation greatly to be desired that the process of rendering a pulp-canal permanently innocuous after the death of the pulp be simplified and made available to a greater number, while at the same time it is rendered less a matter of skill and endurance on the part of the operator. The requirements remain the same as when the knowledge was first acquired that root-canal filling was a cure for the evils following pulp devitalization. We cannot cheat dame nature, although we may find easier ways of complying with her demands.

It has seemed to me that the most promising suggestion of recent years in the direction of simplifying the effectual and permanent treatment of root-canals has been presented to us by Dr. L. P. Bethel. His paper on "Lining Root-Canals"[1] advocates the introduction of silver nitrate into the canals by the aid of the cataphoric action of the galvanic current, and the practicability of the process has been shown to us, from one point of view, by the specimens of teeth whose root-canals were filled with the salt and its compounds, which he kindly sent to one of our meetings. The complete filling of the most tortuous and smallest canals—and even the tubuli of the dentine in some cases—was shown to be entirely feasible. The substance occupying the canals seemed to be a compound of the silver salt with organic matter, and not the salt itself. This point could easily be determined by subjecting such specimens as we saw here to a water-bath; a compound of the salt would probably be insoluble, whereas we know the nitrate of silver itself is readily

---

[1] INTERNATIONAL DENTAL JOURNAL, November, 1896.

soluble. It is just here that the value of Dr. Bethel's suggestion seems to hinge on the peculiar affinity silver nitrate has, and the compounds it forms, with organic matter. This is illustrated when it is applied to a decayed surface of the dentine; the decalcified substance forms a compound with the salt that is so nearly insoluble as to form an efficient protector to the underlying dentine for months or years. None of the gentlemen who criticised Dr. Bethel's method adversely seemed to regard the formation of an insoluble compound in a favorable light: indeed, it was hardly referred to, except to object to its utility; but in this fact, I think, is the great value of silver nitrate, both for the arrest of decay and, I hope, for the complete filling of root-canals. The chemical reaction that takes place when silver nitrate comes into contact with organic matter would of course include all bacteria, and they, as well as everything else organic, would become a part of the new compound, which is probably devoid of germicidal power, but destined, nevertheless, to serve the purpose if it will permanently occupy the space.

It has been noted several times that exposed living and, perhaps, aching pulps are effectually quieted, and may be subsequently removed without pain, if subjected to impregnation with silver nitrate by cataphoresis. I have no doubt that this is the result of the chemical effects of the salt on the nerves, as well as the other pulp-tissue, actually transforming them into a different substance. Like Lot's wife, they are something else before they know it.

I think that what we now know presents a hopeful prospect for the treatment, along this line, of hopeless pulps. If they can be converted into a solid mass of insoluble compound by chemical union with a substance that can be driven into them by the galvanic current, does it not seem as if the requirements for saving pulpless teeth are about to be fulfilled with much less labor than of old, and that the benefits of successful treatment may be greatly extended? The fact that compounds of silver nitrate are so dark in color may operate to limit its use to teeth posterior to the cuspids, but these are the very teeth that most tax our skill and patience, and for whose salvation it is most to be desired that a simpler method may be found. Then it is hardly supposable that this salt will prove to be the only agent that will combine with organic matter in such a way as to form a compound that will serve for the arrest of decay and the occlusion of root-canals.

One of the future advances that we may reasonably hope for is the discovery of an agent that will form a new and stable compound with organic matter, and without serious change of color.

When the chemistry of such reactions, and others which specially concern us, has received a fraction of the attention that has been given to the chemistry of photography, I have no doubt our hopes for the simplification of the processes of saving teeth will be realized.

But even if the necessity of mechanical root-canal filling should be to a great extent obviated, there will still be need of disinfecting root-canals and dentine, and of restoring to health infected tissues around the ends of roots that have contained putrescent pulps.

Disinfection is a chemical and germicidal process, while the treatment of living tissues belongs to therapeutics. I wish to refer briefly to the former process, and discuss a point in the disinfection of root-canals and dentine.

Dr. Black's valuable researches have shown that there is in dentine more than thirty-six per cent. of water and organic matter together, and this shows with great emphasis the importance of disinfecting this tissue, as well as the root-canals themselves, when the space occupied by such a proportion of it is filled with putrescent matter. A quite notable discussion of the means of effecting such disinfection has taken place during the last few years, which will be readily recalled to your minds if I call it the contention for coagulants and for non-coagulants.

Dr. A. W. Harlan has repeatedly expressed the opinion that trouble occurring in pulpless teeth under his observation, whose root-canals had been filled, was due to failure to disinfect the dentine because agents had been used to effect such disinfection that were coagulators of albumen. He claims that coagulating agents are prevented from diffusing through infected matter in tubes by coagulating albumen at the end of the tube with which such disinfectant first comes in contact. This is, I think, a correct statement of Dr. Harlan's objection to the use of coagulants. He has stated the question in the words, "Do coagulating agents prevent their own diffusion through tooth-structure?" Dr. Harlan's position has been endorsed by Dr. Black, and the negative side has been taken by Drs. Truman and Kirk. Both of the latter gentlemen have attempted to prove—and proved, I think—that a coagulum of albumen does not prevent the passage through it of agents causing the coagulation, or that the peripheral extremity of the contents of tubuli are affected by coagulating agents applied to the central ends. There has been much difference of opinion regarding the merits of this controversy, and a number of writers have taken sides pro or con, but there is one point that has appeared to me to have been overlooked,—namely, that the claim, in the first place, that coagulants "prevent their own diffusion through tooth-structure" appears to be based on the apparent effects of coagulants upon normal albumen, and the contention, on the other hand, that the opposite of this statement is true is supported by experiments made also upon normal albumen, or that which has not been acted upon by the micro-organisms of decomposition; while the actual condition under consideration for treatment is that of a tooth whose pulp is dead and putrescent, and the contents of whose dentinal canaliculi have also been disorganized by bacteria. The reason for the need of disinfection of the dentine is that its organic part has become putrescent; what relevancy, then, has the action of coagulants upon normal albumen, when it has not been shown that the action of such agents on putrescent matter is to coagulate it in a similar way to its action on albumen?

Dr. Harlan has admitted that the use of a coagulating antiseptic in a root-canal which has had its pulp removed before putrescence has occurred is not objectionable.[1] It is in the condition that results after "various poisons" have been formed in the dentine tubes that objection is made to the use of coagulating agents; and in support of these objections experiments have been made with albumen, but no proofs have been presented regarding the effects of coagulants on the "various poisons."

The experiments on which Dr. Harlan rests his statements were not, so far as appears, applied to teeth whose dentine was filled with putrescent matter, and no demonstration has been made that disorganized matter is coagulable, as albumen is.

He says,[2] "The test is to introduce the agent into a tooth freshly extracted, from whence the pulp has been removed. . . . The agent was allowed to diffuse or not." There is no mention of the condition of pulp or of dentine in making the tests, but in all references, so far as I know, the argument is based on the action of coagulants on normal albumen. If admission should be made that coagulants prevent their own diffusion through organic matter in a normal state, the action of coagulants upon organic matter changed to a putrescent mass would still remain for consideration. The putrefaction of proteids results in the formation of solids, liquids, and gases, among which are leucin, tyrosin, a number of alkaloids, ptomaines, ammonias, hydrogen sulphide, etc. But it does not appear that any of these in the combinations in which they appear

---

[1] Dental Cosmos, vol. xxxvii., p. 223. [2] Ibid., p. 211.

as the result of putrefaction are coagulable. The efficacy of a disinfecting agent for use in dentine would appear, therefore, to depend upon its chemical action on the mephitic compounds that occupy the tubuli, and upon its germicidal action, and not at all upon its coagulating or non-coagulating properties.

